# Rotavirus disease burden pre-vaccine introduction in young children in Rural Southern Mozambique, an area of high HIV prevalence

**DOI:** 10.1371/journal.pone.0249714

**Published:** 2021-04-08

**Authors:** Sozinho Acácio, Tacilta Nhampossa, Llorenç Quintò, Delfino Vubil, Marcelino Garrine, Quique Bassat, Tamer Farag, Sandra Panchalingam, James P. Nataro, Karen L. Kotloff, Myron M. Levine, Sharon M. Tennant, Pedro L. Alonso, Inácio Mandomando

**Affiliations:** 1 Centro de Investigação em Saúde de Manhiça (CISM), Maputo, Mozambique; 2 Instituto Nacional de Saúde, Ministério da Saúde, Maputo, Mozambique; 3 ISGlobal, Hospital Clínic—Universitat de Barcelona, Barcelona, Spain; 4 Global Health and Tropical Medicine, Instituto de Higiene e Medicina Tropical, Universidade Nova de Lisboa (IHMT, UNL), Lisbon, Portugal; 5 ICREA, Barcelona, Spain; 6 Pediatrics Department, Hospital Sant Joan de Déu (University of Barcelona), Barcelona, Spain; 7 Consorcio de Investigación Biomédica en Red de Epidemiología y Salud Pública (CIBERESP), Madrid, Spain; 8 Center for Vaccine Development and Global Health (CVD), University of Maryland School of Medicine, Baltimore, MD, United States of America; 9 World Health Organization, Geneva, Switzerland; Universita degli Studi di Parma, ITALY

## Abstract

**Background:**

Rotavirus vaccines have been adopted in African countries since 2009, including Mozambique (2015). Disease burden data are needed to evaluate the impact of rotavirus vaccine. We report the burden of rotavirus-associated diarrhea in Mozambique from the Global Enteric Multicenter Study (GEMS) before vaccine introduction.

**Methods:**

A case-control study (GEMS), was conducted in Manhiça district, recruiting children aged 0–59 months with moderate-to-severe diarrhea (MSD) and less-severe-diarrhea (LSD) between December 2007 and November 2012; including 1–3 matched (age, sex and neighborhood) healthy community controls. Clinical and epidemiological data and stool samples (for laboratory investigation) were collected. Association of rotavirus with MSD or LSD was determined by conditional logistic regression and adjusted attributable fractions (AF) calculated, and risk factors for rotavirus diarrhea assessed.

**Results:**

Overall 91***5*** cases and 1,97***7*** controls for MSD, and 431 cases and 43***0*** controls for LSD were enrolled. Rotavirus positivity was 44% (217/495) for cases and 15% (160/1046) of controls, with AF = 34.9% (95% CI: 32.85–37.06) and adjusted Odds Ratio (aOR) of 6.4 p< 0.0001 in infants with MSD compared to 30% (46/155) in cases and 14% (22/154) in controls yielding AF = 18.7%, (95% CI: 12.02–25.39) and aOR = 2.8, p = 0.0011 in infants with LSD. The proportion of children with rotavirus was 32% (21/66) among HIV-positive children and 23% (128/566) among HIV-negative ones for MSD. Presence of animals in the compound (OR = 1.9; p = 0.0151) and giving stored water to the child (OR = 2.0, p = 0.0483) were risk factors for MSD; while animals in the compound (OR = 2.37, p = 0.007); not having routine access to water on a daily basis (OR = 1.53, p = 0.015) and washing hands before cooking (OR = 1.76, p = 0.0197) were risk factors for LSD.

**Conclusion:**

The implementation of vaccination against rotavirus may likely result in a significant reduction of rotavirus-associated diarrhea, suggesting the need for monitoring of vaccine impact.

## Introduction

Despite the decreasing trends observed throughout the last few decades, diarrheal diseases remain among the major causes of morbidity and mortality among children aged 0–59 months worldwide [1]. Rotavirus is still the leading cause of severe dehydration and diarrhea in this age group [2, 3] and was estimated to cause in the year 2013 up to 215,000 deaths, with the majority of these deaths occurring in low and middle income countries (LMICs) [2].

Two live-attenuated oral vaccines, a pentavalent bovine-human reassortant vaccine (RV5; RotaTeq; Merck and Co, Inc. West Point, PA) and a monovalent vaccine based on a human rotavirus strain (RV1; Rotarix; GSK Biological, Rixensart, Belgium), were licensed and have been globally recommended by the World Health Organization (WHO) since the year 2009 for introduction into the national immunization programs of countries, particularly those with high under five diarrheal mortality [3]. Estimated efficacy of these vaccines varied from 49% to 72% in sub-Saharan Africa and Asia [4, 5]. Thus, rotavirus vaccines have been progressively adopted in many countries and are currently implemented in over 100 countries worldwide [6].

In Africa, the first country to introduce rotavirus vaccine was South Africa in the year 2009, with additional countries starting to adopt it in the year 2012, and a much more intense deployment occurring from 2014 onwards [7]. To monitor the impact of vaccine introduction, data documenting the baseline burden of disease preceding its introduction are essential. In Mozambique, epidemiological data on diarrheal disease remain scarce and limited to a few studies [8, 9]. However, two reports analyzing the cause of death using verbal autopsies and complete autopsy in Manhiça and Maputo city, respectively, have documented diarrhea as one of the major causes of death [10, 11]. Rotavirus associated diarrhea has only been reported in a few studies [8, 9], including children seeking care with severe diarrhea. Importantly, only a single study has included adequate controls [8], but the contribution of rotavirus to less severe diarrhea in-country has not been full assessed. We hereby report the burden of rotavirus associated diarrhea among children less than 5 years of age enrolled as part of the Global Enteric Multicenter Study (GEMS) [12], a large and comprehensive multicenter case-control study on the etiology and epidemiology of diarrheal diseases conducted between 2007 and 2012 in Manhiça district. The study was conducted prior to the introduction of the monovalent rotavirus vaccine (Rotarix, GlaxoSmith Kline, Biologicals, Belgium) in September 2015.

## Methods

### Primary study

The GEMS was an age-stratified, case-control study. Children under the age of five years were studied and divided into three age strata: 0–11, 12–23, and 24–59 months. Each of seven sites in Asia and sub-Saharan Africa (Bangladesh, India, Pakistan, Mozambique, Mali, The Gambia, and Kenya) included one to eleven community Sentinel Health Centers (SHCs) serving populations under demographic surveillance [12]. In Mozambique, the study was conducted in the District of Manhiça, a rural area located 80 kilometers north of the capital of Mozambique, Maputo. In this area malaria is endemic with perennial transmission and HIV prevalence is amongst the highest in the world, with prevalence rates in the adult population as high as 40% [13]. During the study period, the Manhiça district population was estimated at 183,000 inhabitants and the *Centro de Investigação em Saúde de Manhiça* (CISM) has been running a demographic surveillance system (DSS) involving intensive and regular monitoring of the entire population in an area covering approximately 92,000 inhabitants [14]. The full characteristics of the study population and research platforms of the CISM have been detailed elsewhere [14].

### Study design

The GEMS was a five-year prospective study to quantify the burden, sequelae and microbiological etiology of moderate-to-severe diarrhea (MSD) (2007 to 2012) [12]. In Manhiça, a specific HIV sub-study was integrated into the generic GEMS protocol in May 2010 and enrollment was extended for an additional 2 years (up to November 2^nd^, 2012), including one-year recruitment of less severe diarrhea (LSD) cases (3^rd^ November 2011 to 2^nd^ November 2012). The analysis presented here refers to five years of MSD cases (December 7^th^, 2007 to November 2^nd^, 2012) and one year of LSD cases (November 3^rd^, 2011 to November 2^nd^, 2012). Children were defined as having diarrhea if they fulfilled the WHO definition of three or more abnormally loose stools per day, and were included as cases if diarrhea represented a new episode (no episodes in preceding 7 days). An MSD-case was defined as a child with diarrhea and at least one of the following: sunken eyes, loss of skin turgor, dysentery, need for intravenous rehydration or hospitalization with diarrhea or dysentery. LSD-cases implied episodes of diarrhea not fulfilling the criteria of moderate-to-severe diarrhea. For each MSD-case one to three matched community controls were randomly selected from the DSS, while only one control was matched for each LSD case. Controls were children with no diarrhea episodes in the 7 days preceding enrolment. Additionally, they were selected on the basis of the following matching criteria: age ± 2 months for age group 0–11 months, ± 4 months for age group 12–59 months, and not exceeding stratum boundaries for the given age group, same gender, residence in same catchment area, and enrolment within 14 days of the index case. Parents or primary caretakers of both cases and controls underwent standardized interviews to collect demographic, epidemiologic, and clinical information. Additionally, anthropometric measurements were taken. GEMS field workers made a single follow-up visit to the household of each case and control child ~60 days after enrollment (range 50–90 days) to assess the child’s vital status, interim medical events, and repeat anthropometric measurements. Further details of clinical and epidemiological design of the GEMS and its Health Utilization and Attitudes Surveys (HUAS), designed to better understand locally-specific health seeking behavior for diarrheal disease, have been described previously [15].

### Laboratory methods for detection of rotavirus

At enrollment, each case and control provided a fresh stool sample collected into a container that was immediately placed in a cold box with icepacks until delivery to the laboratory. If antibiotics were to be administered to the cases before stool collection, two rectal swabs were obtained and placed in transport media (buffered glycerol saline and Cary-Blair) for bacterial culture pending passage of whole stool for the remaining assays.

An extensive workup for a wide array of putative and established viral, bacterial, and protozoan diarrheal pathogens was undertaken on each case and control specimen. A commercial immunoassay was used for the detection of rotavirus antigens in stool (ProSpecT Rotavirus kit, Oxoid, Basingstoke, UK). Other pathogens were assessed using standard microbiological and molecular techniques as described elsewhere [16]. A subset of positive samples by ELISA (n = 158) were retested by TaqMan Array Cards-TAC (Thermo Fischer, Carlsblad, CA, USA) [17], and genotyped by conventional multiplex polymerase chain reaction (PCR) as previously described [18]. Samples were considered positive for rotavirus if they tested positive either by ELISA, conventional PCR or TAC.

## Statistical analysis

Analyses were conducted using Stata/SE software version 14.1 [19] and the package coxphf from R, version 3.2.2 [20] stratified by age as per sampling frame. Ordinary or conditional logistic regression models were used to evaluate associations, depending on the issue of analysis. All these models were estimated with the penalized likelihood according to the Firth’s approach. Multivariable models were estimated by forward-stepwise selection from covariates with p<0.20 in the crude models and no more than 5% of missing values. Significant levels for removal and addition in the stepwise procedure were 0.10 and 0.05 respectively by Wald test. Analysis of the association between MSD or LSD and rotavirus were performed by conditional logistic regression, at crude level for all pathogens and for rotavirus adjusted for other significant pathogens and pairwise-interactions between each one of them with rotavirus.

Population attributable fractions of MSD/LSD (unadjusted and adjusted), annual attributable cases and attributable incidences were calculated. According to the study protocol, cases of MSD/LSD were included in approximately equal numbers each fortnight, regardless of the number of cases visited in the Sentinel Health Centers (SHC). This was taken into account and weighted attributable fractions were also estimated, using weights defined as the inverse of the sampling fraction (number of eligible cases divided by the number of enrolled cases in each fortnight) [21]. These weights were calculated separately for cases with and without dysentery, to avoid any bias from overrepresentation or underrepresentation of cases with dysentery. We combined data for two or more adjacent fortnights to have at least one case with dysentery and at least one case without dysentery in each time period. Unadjusted/adjusted, weighted/unweighted attributable fractions (AF) were calculated as in Bruzzi et al. for all variables with a positive association with MSD/LSD [22]. The variance of AF was approximated by Taylor series to first derivative terms (delta method) [23, 24].

To estimate the burden of disease, repeated surveys were conducted during the case-control study to find out the proportion of cases that usually goes to the SHC within one week of onset of MSD/LSD (called r). These surveys were conducted on random samples of children, every 6 months with each round of the DSS. We combined the data from these surveys and we weighted them by sampling weights, based on the number of children in each age-sex stratum according to information from DSS at the time of the round [21]. After that, we estimated the values of r and its variances for each age stratum by Kaplan Meier analysis.

The annual number of cases of MSD/LSD in the population was calculated as the average number of eligible cases per year (total eligible divided by 5) divided by r. The annual cases divided by the median of the population gave the MSD/LSD incidence rate. To calculate the number of cases and the incidence rates attributable to rotavirus, the total cases and incidence rates were multiplied by the rotavirus’ weighted and adjusted AF [21]. The variance of the incidence rate was approximated by Taylor series to first derivative terms (delta method) [23, 24]. The variance of the number of eligible cases coming to the SHC was estimated as the variance of a Binomial distribution with n = total number of children visited at the SHC during the 5 years of surveillance and p = proportion of eligible [25]. To estimate the median population we used the DSS information from all rounds conducted during the case-control study and we estimated the variance as the variance of the median of several observations from a normal distribution [26, 27]. The variance of r and the variance of AF were estimated as described above.

Analysis of socio-demographic factors associated with rotavirus infection was done separately between cases and controls, since the study design did not allow us to assess such associations in the entire sample for the outcomes other than being a MSD case. Analysis of signs and symptoms associated with rotavirus-MSD *versus* other types of MSD was performed among MSD-cases, by ordinary logistic regression models. Interactions between significant covariates in the multivariable model were also assessed. Furthermore, we investigated the association between dysentery and rotavirus (only for MSD cases).

## Ethical statement

This is a sub-analysis deriving from the Global Enteric Multicenter Study (GEMS). The overall GEMS protocol and informed consent were both approved by the National Bioethics Committee for Health of Mozambique (**CNBS–IRB00002657**), the ethics committee of the Hospital Clinic of Barcelona and the Institutional Review Board at the University of Maryland. After informing the objectives and characteristics of the study a written informed consent was obtained from the child’s caretaker. One copy of the consent form was left with the caretaker and the other retained in locked cabinets at CISM.

## Results

During the study period, a total of 915 MSD cases and 1,977 matched controls (December 2007 to November 2^nd^ 2012); and 431 LSD cases and their respective 430 matched controls (November 3^rd^ 2011 to November 2^nd^ 2012) were enrolled. Among infants with MSD, rotavirus was detected in 44% (217/495) of the cases and 15% (160/1,046) of controls, giving an AF of 34.9% (95% CI: 32.85; 37.06) and an adjusted Odds Ratio (aOR) of 6.4 (95% CI: 4.69–8.79; p< 0.0001). The rate of rotavirus detection as well as AF decreased with age **(Tables 1 and 2)**. Similarly, rotavirus was more common among infants with LSD with a detection rate of 30% (46/155) in cases and 14% (22/154) in controls, yielding an AF of 24.0%, (95% CI: 17.79; 30.18) and an aOR of 2.8 [95%CI: 1.53; 5.41, p = 0.0011] **(Tables 1 and 2)**. Interestingly, the incidence rate of rotavirus was much higher among infants with LSD reaching 24.7 (95% CI: 15.02–34.35) per 100 children-years-at-risk compared to MSD 3.07 (95% CI: 2.49–3.66) per 100 children-years-at-risk in the same age group, as shown in **(Table 3)**.

**Table 1:** Prevalence of rotavirus among children 0–59 months of age with diarrhea (MSD & LSD) and their matched controls.

Age group	Group			
	Controls	Cases	Total	*p*-value
**MSD**				
**0–11 months**	160/1046 (15%)	217/495 (44%)	377/1541 (24%)	< 0.0001
**12–23 months**	102/628 (16%)	62/275 (23%)	164/903 (18%)	0.0014
**24–59 months**	33/303 (11%)	16/145 (11%)	49/448 (11%)	0.4999
**Overall**	**295/1977 (15%)**	**295/915 (32%)**	**590/2892 (20%)**	
**LSD**				
**0–11 months**	22/154 (14%)	46/155 (30%)	68/309 (22%)	0.0012
**12–23 months**	35/175 (20%)	21/175 (12%)	56/350 (16%)	0.0436
**24–59 months**	17/101(17%)	13/101(13%)	30/202(15%)	0.5572
**Overall**	**74/430 (17%)**	**80/431 (19%)**	**150/861 (17%)**	

**Table 2:** Attributable fractions (AF) of rotavirus among children 0–59 months with MSD and LSD cases.

Age group	Unadjusted Analysis	Adjusted Analysis		
	AF (95% CI)	AF (95% CI)	aOR (95% CI)	*p*-value
**MSD cases**				
**0–11 months**	34.9 (32.85; 37.06)	34.8 (31.74; 38.03)	6.4 (4.69, 8.79)	< 0.0001
**12–23 months**	12.0 (6.74; 17.32)	12.7 (4.60; 20.85)	2.1 (1.36, 3.16)	0.0007
**24–59 months**	-	-	-	-
**LSD cases**				
**0–11 months**	18.7 (12.02; 25.39)	24.0 (17.79; 30.18)	2.8 (1.53; 5.41)	0.0011
**12–23 months**	11.8 (4.25; 19.44)	-	-	-
**24–59 months**	**-**	**-**	**-**	**-**

**Table 3:** Rotavirus incidence rate by age group among MSD and LSD cases.

Age group		MSD rate per 100 children-years-at-risk (CY)	
**MSD cases**	**Annual Cases**	**Estimation**	**95% Conf. Interval**
**0–11 months**	99	3.07	(2.49; 3.66)
**12–23 months**	29	0.91	(0.30; 1.52)
**24–59 months**	-	-	**-**
**LSD cases**		**LSD rate per 100 children-years-at-risk (CY)**	
**0–11 months**	702	24.68	(15.02; 34.35)
**12–23 months**	-	**-**	**-**
**24–59 months**	-	**-**	**-**

A subset of 158 rotavirus ELISA positive samples were retested, and 57.6% (n = 91) and 58.2% (n = 92) were positive for TAC and PCR, respectively. Fourth-one percent (n = 65) of the samples were simultaneously TAC and PCR positives; 16% (n = 26) and 17% (n = 26) were exclusively positive for PCR and TAC, respectively. The remaining twenty-five percent were negative for both methods.

### Clinical presentation and risk factors

The odds of having rotavirus in children with MSD was higher among those with severe signs and symptoms such as sunken eyes, loss of skin turgor, vomiting three or more times per day, wrinkled skin, dry mouth, long or very long skin pinch and the need for intravenous rehydration **(Table 4)**.

**Table 4:** Signs and symptoms of children 0–59 months of age with moderate-to-severe diarrhea associated with confirmed rotavirus. (Crude and multivariate estimation).

Variable	Unadjusted				Adjusted	
	Type of MSD					
	Other MSD	Rotavirus MSD	OR (95% CI)	*p*-value	aOR (95% CI)	*p*-value
**0–11 months**						
Sunken eyes	123/278 (44%)	159/217 (73%)	3.4 (2.34, 5.03)	< 0.0001	3.0 (2.01, 4.47)	< 0.0001
Loss of skin turgor	77/278 (28%)	97/217 (45%)	2.1 (1.45, 3.06)	0.0001		
Intravenous rehydration	125/278 (45%)	137/217 (63%)	2.1 (1.45, 3.00)	0.0001		
Hospitalized	202/278 (73%)	147/217 (68%)	0.8 (0.54, 1.16)	0.2334		
Vomiting 3 or more times per day	122/278 (44%)	146/217 (67%)	2.6 (1.81, 3.78)	<0.0001	2.1 (1.40, 3.06)	0.0003
Drank much less than usual	49/278 (18%)	22/217 (10%)	0.5 (0.31, 0.91)	0.0210		
Unable to drink	31/278 (11%)	12/217 (6%)	0.5 (0.24, 0.94)	0.0335		
Belly pain	72/278 (26%)	59/217 (27%)	1.1 (0.72, 1.60)	0.7432		
Fever	111/278 (40%)	61/217 (28%)	0.6 (0.40, 0.86)	0.0066		
Irritable or restless	43/277 (16%)	42/217 (19%)	1.3 (0.82, 2.08)	0.2620		
Decreased activity or lethargy	129/277 (47%)	110/217 (51%)	1.2 (0.83, 1.68)	0.3640		
Loss of consciousness	4/278 (1%)	4/217 (2%)	1.3 (0.34, 4.81)	0.7088		
Rectal prolapse	2/277 (1%)	0/217 (0%)	0.3 (0.01, 5.30)	0.3763		
Convulsion	9/278 (3%)	4/217 (2%)	0.6 (0.19, 1.86)	0.3749		
Very thirsty	188/277 (68%)	165/216 (76%)	1.5 (1.02, 2.28)	0.0391		
Drinks poorly	43/277 (16%)	18/217 (8%)	0.5 (0.28, 0.89)	0.0183		
*Wrinkled skin	74/277 (27%)	92/217 (42%)	2.0 (1.38, 2.94)	0.0003		
Lethargy or loss of consciousness	128/278 (46%)	111/217 (51%)	1.2 (0.86, 1.75)	0.2601		
Dry mouth	97/277 (35%)	120/217 (55%)	2.3 (1.59, 3.29)	< 0.0001		
Fast breathing	92/278 (33%)	57/217 (26%)	0.7 (0.49, 1.07)	0.1030		
Long or very long skin pinch	66/259 (25%)	79/196 (40%)	1.9 (1.32, 2.93)	0.0009		
**12–23 months**						
Sunken eyes	88/213 (41%)	39/62 (63%)	2.4 (1.34, 4.25)	0.0033	2.6 (1.43, 4.79)	
Loss of skin turgor	45/213 (21%)	25/62 (40%)	2.5 (1.38, 4.59)	0.0026		
Intravenous rehydration	84/213 (39%)	36/62 (58%)	2.1 (1.19, 3.73)	0.0103		
Hospitalized	143/213 (67%)	41/62 (66%)	0.9 (0.52, 1.72)	0.8609		
Vomiting 3 or more times per day	92/213 (43%)	39/62 (63%)	2.2 (1.24, 3.93)	0.0072	1.9 (1.05, 3.44)	
Drank much less than usual	12/213 (6%)	3/62 (5%)	0.9 (0.28, 3.21)	0.9320		
Unable to drink	14/213 (7%)	3/62 (5%)	0.8 (0.24, 2.69)	0.7303		
Belly pain	55/213 (26%)	19/62 (31%)	1.3 (0.69, 2.37)	0.4315		
Fever	74/213 (35%)	19/62 (31%)	0.8 (0.46, 1.54)	0.5697		
Irritable or restless	30/213 (14%)	10/62 (16%)	1.2 (0.56, 2.59)	0.6356		
Decreased activity or lethargy	110/213 (52%)	34/62 (55%)	1.1 (0.65, 1.99)	0.6624		
Loss of consciousness	2/213 (1%)	3/62 (5%)	4.9 (0.96, 25.87)	0.0564		
Rectal prolapse	0/213 (0%)	0/62 (0%)				
Convulsion	11/213 (5%)	2/62 (3%)	0.7 (0.18, 2.94)	0.6557		
Very thirsty	172/213 (81%)	51/62 (82%)	1.1 (0.52, 2.22)	0.8400		
Drinks poorly	12/212 (6%)	4/62 (6%)	1.2 (0.40, 3.77)	0.7121		
Wrinkled skin	41/213 (19%)	23/62 (37%)	2.5 (1.34, 4.56)	0.0038		
Lethargy or loss of consciousness	98/211 (46%)	36/62 (58%)	1.6 (0.90, 2.80)	0.1112		
Dry mouth	88/213 (41%)	32/62 (52%)	1.5 (0.86, 2.66)	0.1514		
Fast breathing	41/212 (19%)	14/61 (23%)	1.3 (0.64, 2.49)	0.5024		
Low or very low skin pinch	34/201 (17%)	20/54 (37%)	2.9 (1.49, 5.57)	0.0016		
**24–59 months**						
Sunken eyes	46/129 (36%)	5/16 (31%)	0.9 (0.29, 2.52)	0.7819		
Loss of skin turgor	18/129 (14%)	4/16 (25%)	2.2 (0.66, 7.09)	0.1999		
Intravenous rehydration	36/129 (28%)	3/16 (19%)	0.7 (0.19, 2.29)	0.5164		
Hospitalized	56/129 (43%)	8/16 (50%)	1.3 (0.47, 3.58)	0.6104		
Vomiting 3 or more times per day	33/129 (26%)	8/16 (50%)	2.9 (1.03, 8.06)	0.0438	6.1 (1.82, 20.63)	0.0034
Drank much less than usual	12/129 (9%)	0/16 (0%)	0.3 (0.02, 5.04)	0.3917		
Unable to drink	12/129 (9%)	0/16 (0%)	0.3 (0.02, 5.04)	0.3917		
Belly pain	30/129 (23%)	3/16 (19%)	0.0 (0.24, 2.93)	0.7918		
Fever	47/128 (37%)	4/16 (25%)	0.6 (0.20, 1.92)	0.4056		
Irritable or restless	10/129 (8%)	1/ 15 (7%)	1.2 (0.20, 7.09)	0.8586	7.2 (1.26, 40.64)	0.0261
Decreased activity or lethargy	65/129 (50%)	3/15 (20%)	0.3 (0.08, 0.95)	0.0407	0.1 (0.03, 0.59)	0.0070
Loss of consciousness	3/129 (2%)	0/16 (0%)	1.1 (0.05, 22.16)	0.9527		
Rectal prolapse	1/129 (1%)	0/16 (0%)	2.6 (0.10, 66.38)	0.5641		
Convulsion	8/129 (6%)	1/16 (6%)	1.4 (0.23, 8.49)	0.7260		
Very thirsty	102/129 (79%)	14/16 (88%)	1.6 (0.38, 6.35)	0.5378		
Drinks poorly	16/129 (12%)	0/16 (0%)	0.2 (0.01, 3.64)	0.2826		
Wrinkled skin	18/129 (14%)	3/16 (19%)	1.6 (0.44, 5.59)	0.4925		
Lethargy or loss of consciousness	58/129 (45%)	3/16 (19%)	0.3 (0.09, 1.08)	0.0660		
Dry mouth	36/129 (28%)	4/16 (25%)	0.9 (0.29, 2.89)	0.8896		
Fast breathing	8/129 (6%)	0/16 (0%)	0.4 (0.02, 7.86)	0.5715		
Low or very low skin pinch	14/120 (12%)	3/16 (19%)	1.9 (0.52, 6.97)	0.3305		

Table 5 describes the socio-demographic factors for rotavirus infection among MSD-cases. Among MSD-cases only children less than one year of age had risk factors associated with rotavirus infection, which included: Giving stored water to the child OR = 2.0 (95% CI:1.01–4.15, p = 0.048), the presence of animals in the compound OR = 1.8 (95% CI: 1.13–3.11, p = 0.015), and partial or exclusive breastfeeding OR = 5.8 (95% CI: 1.06, 32.47, p = 0.043). Water availability (not always per day), not having routine access to water on a daily basis seemed to protect against rotavirus infection, OR = 0.5 (95% CI: 0.40–0.81, p = 0.0019) **(Table 5)**.

**Table 5:** Socio-demographic factors associated with rotavirus infections among children 0–59 months of age with moderate-to-severe diarrhea.

Variable	Unadjusted				Adjusted	
	Rotavirus					
	Positive	Negative	OR (95%CI)	p-value	aOR (95%CI)	p-value
**0–11 months**						
Child primary caretaker (Mother)	272/278 (98%)	207/217 (95%)	0.5 (0.17, 1.27)	0.1384		
Caretaker formal education	68/276 (25%)	70/217 (32%)	1.5 (0.98, 2.16)	0.0620		
Animals in compound	223/278 (80%)	192/217 (88%)	1.9 (1.13, 3.11)	0.0151	1.7 (1.03, 2.88)	0.0388
Water availability (not always per day)	163/277 (59%)	97/217 (45%)	0.6 (0.40, 0.81)	0.0019	0.6 (0.41, 0.86)	0.0050
Access to improved water	235/278 (85%)	180/217 (83%)	0.9 (0.55, 1.43)	0.6297		
Give stored water to child	250/278 (90%)	206/217 (95%)	2.0 (1.01, 4.15)	0.0483		
Treating water habit	30/278 (11%)	25/217 (12%)	1.1 (0.62, 1.89)	0.7889		
Facility to dispose child’s stool	138/276 (50%)	112/216 (52%)	1.1 (0.75, 1.54)	0.6842		
Improved facility for household stool	24/278 (9%)	19/216 (9%)	1.0 (0.55, 1.91)	0.9366		
Wash hands before eating	266/278 (96%)	202/217 (93%)	0.6 (0.28, 1.32)	0.2109		
Wash hands before cooking	164/278 (59%)	143/217 (66%)	1.3 (0.93, 1.94)	0.1181		
Wash hands before preparing food	71/278 (26%)	61/217 (28%)	1.1 (0.77, 1.70)	0.5186		
Wash hands after defecating	234/278 (84%)	185/217 (85%)	1.1 (0.66, 1.77)	0.7502		
Wash hands after handling animals	5/278 (2%)	7/217 (3%)	1.2 (0.58, 5.40)	0.3146		
Wash hands after cleaning child feces	65/278 (23%)	64/217 (29%)	1.4 (0.92,2.05)	0.1247		
Partial or exclusive breastfeeding	237/248 (96%)	181/182 (99%)	5.9 (1.06, 32.47)	0.0430		
**12–23 Months**						
Child primary caretaker (Mother)	195/213 (92%)	58/62 (94%)	1.2 (0.42, 3.59)	0.7046		
Caretaker formal education	46/212 (22%)	16/621 (26%)	1.3 (0.68, 2.49)	0.4312		
Animals in compound	176/213 (83%)	5/62 (84%)	1.1 (0.50, 2.25)	0.8745		
Water availability (not always per day)	117/213 (55%)	34/62 (55%)	0.9 (0.57, 1.75)	0.9838		
Access to improved water	175/213 (82%)	54/62 (87%)	1.4 (0.63, 3.14)	0.4048		
Give stored water to child	208/213 (98%)	59/62 (95%)	0.5 (0.11, 1.77)	0.2515		
Treating water habit	12/213 (6%)	4/62 (6%)	1.2 (0.41, 3.79)	0.7056		
Facility to dispose child’s stool	173/212 (82%)	51/61 (84%)	1.1 (0.53, 2.36)	0.7726		
Improved facility for household stool	11/213 (5%)	4/62 (6%)	1.4 (0.44, 4.18)	0.5980		
Wash hands before eating	188/213 (88%)	55/62 (89%)	1.0 (0.42, 2.38)	0.9981		
Wash hands before cooking	129/213 (61%)	34/62 (55%)	0. 8 (0.45, 1.39)	0.4147		
Wash hands before preparing food	59/213 (28%)	23/62 (37%)	1.5 (0.85, 2.79)	0.1497		
Wash hands after defecating	185/213 (87%)	52/62 (84%)	0. 8 (0.36, 1.66)	0.5026		
Wash hands after handling animals	3/213 (1%)	1/62 (2%)	1.5 (0.21,10.14)	0.6978		
Wash hands after cleaning child feces	50/213 (23%)	16/62 (26%)	1.2 (0.60, 2.19)	0.6729		
Partial or exclusive breastfeeding	104/179 (58%)	37/52 (71%)	1. 8 (0.90, 3.39)	0.0981		
**24–59 Months**						
Child primary caretaker (Mother)	114/129 (88%)	14/16 (88%)	0. 8 (0.19, 3.32)	0.7425		
Caretaker formal education	30/128 (23%)	4/16 (25%)	1.2 (0.37, 3.68)	0.7976		
Animals in compound	103/129 (80%)	16/16 (100%)	8.5 (0.49,145.44)	0.1416		
Water availability (not always per day	59/129 (46%)	8/16 (50%)	1.2 (0.43, 3.26)	0.7424		
Access to improved water	106/129 (82%)	14/16 (88%)	1.3 (0.31, 5.27)	0.7325		
Give stored water to child	122/128 (95%)	14/16 (88%)	0.3 (0.06, 1.46)	0.1379		
Treating water habit	8/129 (6%)	0/16 (0%)	0.4 (0.02, 7.86)	0.5715		
Facility to dispose child’s stool	123/129 (95%)	15/16 (94%)	0.5 (0.09, 3.47)	0.5193		
Improved facility for household stool	15/129 (12%)	2/16 (12%)	1.3 (0.30, 5.39)	0.7425		
Wash hands before eating	117/129 (91%)	16/16 (100%)	3.5 (0.20, 62.12)	0.3917		
Wash hands before cooking	79/129 (61%)	10/16 (62%)	1.0 (0.36, 2.90)	0.9612		
Wash hands before preparing food	34/129 (26%)	3/16 (19%)	0.7 (0.21, 2.48)	0.5995		
Wash hands after defecating	111/129 (86%)	14/16 (88%)	1.0 (0.23, 4.02)	0.9580		
Wash hands after handling animals	5/129 (4%)	0/16 (0%)	0.1 (0.04, 12.98)	0.8016		
Wash hands after cleaning child feces	24/129 (19%)	5/16 (31%)	2.1 (0.68, 6.23)	0.2009		
Partial or exclusive breastfeeding	18/108 (17%)	4/12 (33%)	2.6 (0.74, 9.01)	0.1348		

In the control group, the presence of animals in the compound OR = 2.4 (95% CI: 1.27, 4.43, p = 0.007); water availability (not always per day) OR = 1.5 (95% CI: 1.08, 2.16, p = 0.015) and washing hands before cooking OR = 1.7 (95% CI: 1.07, 2.91, p = 0.0197) were all risk factors associated with rotavirus infection among children less than one year of age (0–11 months). Washing hands after defecating OR = 1.8 (95% CI: 1.11, 3.24, p = 0.0197) was associated with rotavirus in children aged 12–23 months. Water availability (not always per day) OR = 2.2 (95% CI: 1.10, 4.64, p = 0.026) was the only risk factor associated with rotavirus infection in children aged 24–59 months.

As HIV is highly prevalent in the community studied, we assessed rotavirus positivity according to HIV status, documenting 32% (21/66) of rotavirus detection among HIV-infected children, compared with 23% (128/566) among those not infected with HIV in both groups (cases and controls) in the MSD group. For the LSD group, the prevalence of rotavirus was 12% (5/40) among HIV-infected children and 20% (104/523) among HIV-negative children also in both groups cases and controls ***(Table 6)***.

**Table 6:** Distribution of rotavirus infection in children with MSD and LSD according to HIV status cases and controls.

Variable	Group		
	Controls	Cases	Total
**Moderate to Severe Diarrhea (MSD)**			
Rotavirus among HIV-negatives	62 / 401 (15%)	66 / 165 (40%)	128 / 566 (23%)
Rotavirus among HIV-positives	4 / 17 (24%)	17 / 49 (35%)	21 / 66 (32%)
***Rotavirus—(OVERALL) ***	**66 / 418 (16%)**	**83 / 214 (39%)**	**149 / 632 (24%)**
**Less Severe Diarrhea (LSD)**			
Rotavirus among HIV-negatives	21 / 200 (10%)	83 / 323 (26%)	104 / 523 (20%)
Rotavirus among HIV-positives	2 / 14 (14%)	3 / 26 (12%)	5 / 40 (12%)
***Rotavirus—(OVERALL) ***	**23 / 214 (11%)**	**86 / 349 (25%)**	**109 / 563 (19%)**

## Discussion

This study highlights the significance and magnitude of the rotavirus burden among Mozambican children, not only as a major cause of MSD, but also of LSD. Data generated in the Manhiça district during the GEMS project regarding rotavirus burden was pivotal for supporting the countries’ application to the Vaccine Alliance (GAVI) for the national introduction of rotavirus vaccine as part of the Expanded Program of Immunization. Rotavirus surveillance is currently ongoing to evaluate the impact of vaccine introduction, with an expected significant decrease in diarrheal disease burden.

The rotavirus-associated burden found in this study was significant. Indeed, over a third of all MSD cases could be attributable to rotavirus, whereas nearly a quarter of all LSD cases were also attributable to this pathogen. It is particularly interesting to highlight in how much the effect of rotavirus was among infants, marking this being a vulnerable group that suffered most from the impact of rotavirus transmission. In these five years’ worth of data, we again confirmed that the burden of rotavirus-associated MSD was higher in Mozambique, in comparison to any other GEMS sites [21, 28]. Interestingly, the attributable fraction of rotavirus for LSD found in Mozambican infants is comparable to those reported for MSD in some of the other GEMS sites [21]. Furthermore, the high detection rate of rotavirus among infants with LSD (around 30%) may partly explain the high rate of asymptomatic carriage observed in Mozambican children with no diarrhea (children enrolled as controls in the GEMS) as previously reported in the MSD study [8]. This finding could reflect the high health-seeking behavior of Manhiça’s communities, whereby approximately 85% of responders to the Health Care Services Utilization and Attitudes Survey **(**HUAS) reported seeking health assistance within the first 48 hours of disease onset [15]. In addition, discrepancies between TAC and ELISA generated laboratory data can be explained by the genetic diversity of rotavirus strains circulating in Mozambique as strains from animal origin were previously documented in our setting [29, 30], phenomena also observed in other African countries [31, 32].

Interestingly, for LSD with the exception of infants, where rotavirus continued to be a significant risk factor for LSD, in the older ages rotavirus tended to be more frequently found among controls than cases (Table 1). This is likely to occur because after the first year of life, the child has widened its diet including other foods besides breastfeeding, and particularly other fluids that may be contaminated by rotavirus. In addition, the high incidence rate of rotavirus among LSD cases may reinforce the need for continuous sensitization of the population in relation to adequate WASH practices and for prompt utilization of health system in case of diarrheal disease particularly in infants.

As expected, the signs and/or symptoms of severity among children infected by rotavirus associated with MSD were mainly those related to severe dehydration, including sunken eyes, loss of skin turgor, vomiting three or more times per day and being irritable or restless, which are common features of rotavirus infections [33, 34]. Clinicians should therefore be aware that severity among diarrhea cases in this setting is not necessarily only linked to bacterial pathogens.

In addition, we were able to demonstrate that some socio demographic characteristics and WASH practice seem to play an important role as risk factors for rotavirus infection and MSD. The fact that stored water consumption was associated with rotavirus infection leads us to believe that the hygienic conditions of containers where the water is stored are not adequate, or that the time of water storage may be excessive. Water containers, if not properly cleaned, can function as reservoirs of pathogenic agents and serve as a source of transmission of enteric pathogens. Surprisingly, washing hands before eating and washing hands after cleaning the child’s feces appeared to also be associated with rotavirus infection, something that could be linked to an inadequate hand-washing process, or to contamination of the water used for hand washing.

Conversely, the fact that the unavailability of water throughout the day was not associated with rotavirus infection may reflect the rational use of the scarce available water at the household level; or be linked to the hypothesis that the lack of water can interrupt the chain of transmission of some waterborne transmitted enteropathogens like rotavirus.

The finding of the existence of animals in the yard to be associated with rotavirus infection in both cases and controls especially in young children aged 0–11 months may suggest the possible presence of zoonotic strains [29, 30]. However, and despite other studies supporting the existence of rotavirus strains that infect domestic and wild animals including birds and mammals [35, 36], we were unable to draw more robust conclusions on this regard, as no animal samples were collected. In this respect, previous studies have reported the existence of strains that infect animals and humans causing diarrhea [32, 37].

The high rate of asymptomatic rotavirus carriage found in this study was surprising, exceeding the commonly reported ~5% level. The interpretation of these data is challenging, and such a finding is a matter of concern and may require further investigation. We, however, first ruled out the possibility of misdiagnosis by employing molecular techniques (RT-PCR) on those samples that were positive by ELISA. A possible explanation may relate to the high HIV prevalence in our study setting (estimated at 24.5% among children with MSD and 6.4% in controls [38] and to the likely circulation of zoonotic strains as supported by previous studies [29–31]. Additionally, similar rates of asymptomatic carriage are currently being detected in our ongoing post-GEMS diarrheal disease surveillance, in place since September 2015 (unpublished data). Very few previous studies have reported similarly high rates of rotavirus detection among healthy children [39]. These variations in the frequency of asymptomatic carriage may be a consequence of general conditions of hygiene and different strains of circulating virus. Therefore diarrhea may be the result of an infection with a different genotype or some sort of combination of host and virus factors.

The high burden of rotavirus reported in this study, together with data generated in the urban area of Maputo were critical to inform Mozambique’s Ministry of Health, and for the subsequent deployment of the rotavirus vaccine in September 2015 as part of a comprehensive approach for the prevention and control of pediatric diarrheal disease [40].

## Conclusion

We demonstrated a high burden of rotavirus-associated diarrhea, particularly among infants seeking care for both MSD and LSD. These data supported the long-awaited introduction of the rotavirus vaccine in Mozambique and warrants the continuous surveillance of the etiology and trends of diarrheal disease in the country.
